# Nanoformulation of Geranylgeranyltransferase-I Inhibitors for Cancer Therapy: Liposomal Encapsulation and pH-Dependent Delivery to Cancer Cells

**DOI:** 10.1371/journal.pone.0137595

**Published:** 2015-09-09

**Authors:** Jie Lu, Kohei Yoshimura, Koichi Goto, Craig Lee, Ken Hamura, Ohyun Kwon, Fuyuhiko Tamanoi

**Affiliations:** 1 Dept. of Microbiology, Immunology and Molecular Genetics, Jonsson Comprehensive Cancer Center, University of California Los Angeles, Los Angeles, CA 90095, United States of America; 2 DDS Research Laboratory, NOF CORPORATION, Kawasaki, Kanagawa 210–0865, Japan; 3 Division of Applied Life Science, Graduate School of Engineering, Sojo University, Kumamoto, Japan; 4 Dept. of Chemistry and Biochemistry, University of California Los Angeles, Los Angeles, CA 90095, United States of America; University of Nebraska Medical Center, UNITED STATES

## Abstract

Small molecule inhibitors against protein geranylgeranyltransferase-I such as P61A6 have been shown to inhibit proliferation of a variety of human cancer cells and exhibit antitumor activity in mouse models. Development of these inhibitors could be dramatically accelerated by conferring tumor targeting and controlled release capability. As a first step towards this goal, we have encapsulated P61A6 into a new type of liposomes that open and release cargos only under low pH condition. These low pH-release type liposomes were prepared by adjusting the ratio of two types of phospholipid derivatives. Loading of geranylgeranyltransferase-I inhibitor (GGTI) generated liposomes with average diameter of 50–100 nm. GGTI release in solution was sharply dependent on pH values, only showing release at pH lower than 6. Release of cargos in a pH-dependent manner inside the cell was demonstrated by the use of a proton pump inhibitor Bafilomycin A1 that Increased lysosomal pH and inhibited the release of a dye carried in the pH-liposome. Delivery of GGTI to human pancreatic cancer cells was demonstrated by the inhibition of protein geranylgeranylation inside the cell and this effect was blocked by Bafilomycin A1. In addition, GGTI delivered by pH-liposomes induced proliferation inhibition, G1 cell cycle arrest that is associated with the expression of cell cycle regulator p21^CIP1/WAF1^. Proliferation inhibition was also observed with various lung cancer cell lines. Availability of nanoformulated GGTI opens up the possibility to combine with other types of inhibitors. To demonstrate this point, we combined the liposomal-GGTI with farnesyltransferase inhibitor (FTI) to inhibit K-Ras signaling in pancreatic cancer cells. Our results show that the activated K-Ras signaling in these cells can be effectively inhibited and that synergistic effect of the two drugs is observed. Our results suggest a new direction in the use of GGTI for cancer therapy.

## Introduction

A class of anticancer drugs intended to inhibit membrane association of signaling proteins have been developed over the years. GGTI (geranylgeranyltransferase-I inhibitor) exemplifies this type of anticancer drugs [1–3]. GGTI inhibits protein geranylgeranyltransferase I (GGTase-I), an enzyme that adds a C20 geranylgeranyl group to proteins such as RhoA, RhoC, Rap1 and Ral at the cysteine within the carboxy-terminal tetrapeptide consensus sequence CAAL (C is cysteine, A is an aliphatic amino acid, and the C-terminal residue is leucine or phenylalanine). Characterization of mice with conditional knockout of GGTase-I showed that the GGTase-I deficiency results in the inhibition of oncogenic K-ras-induced lung tumor formation and dramatically increases survival of mice [4]. GGTase-I inhibition results in proliferation inhibition associated with G1 arrest and accumulation of cell cycle regulators such as p21^CIP1/WAF1^, pointing to the importance of GGTase-I in cell proliferation and cell cycle progression [5–7].

By screening a chemical compound library constructed by phosphine catalysis of allenoate compounds, we previously identified several GGTase-I inhibitor (GGTI) compounds that block the protein modification and inhibit membrane association and function of Ral, Rho, and Rap subfamily proteins [8,9]. These compounds inhibit GGTase-I by competing with its substrate proteins. Cell active compounds P61A6 and P61E7 caused cell cycle arrest and suppressed the growth of human cancer cell lines including pancreatic cancer and non-small cell lung cancer [10,11]. Efficacy of GGTI P61A6 to inhibit tumor growth was demonstrated using human pancreatic cancer xenograft [10]. In this experiment, significant inhibition of tumor growth was observed with little side effects as judged by kidney and liver enzyme profiles and by hematologic characterization. Inhibition of geranylgeranylation in the tumor was demonstrated. A similar inhibition of tumor growth was observed by the use of lung cancer xenografts in mice [11].

A major challenge for further GGTI development is to confer tumor targeting capability to these compounds. While it is possible to use low amounts of GGTI to minimize potential side effects, the possibility that there is dose-limiting toxicity of this GGTI compound cannot be discounted, since GGTase-I is an enzyme that functions also in normal cells. Thus, it is important to develop a new generation of nano-formulated GGTI that preferentially delivers GGTI compound to tumors. This would enable tumor targeting, decrease undesirable distribution to other parts of the body, thus avoiding any potential effects on normal tissues.

A dramatic advance in Nanotechnology has led to the development of a number of drug delivery systems including liposomes, polymer micelles, viruses and mesoporous silica nanoparticles [12–25]. These nanoparticles can deliver the drug to tumor by enhanced permeability and retention effect [13] as well as by the use of ligands that target receptors on cancer cells, thus avoiding undesirable systemic chemotoxicity. Among them, liposomes have advantageous features that include efficient encapsulation, relatively easy preparation, biodegradability and biocompatibility [26–31]. In addition, the phospholipid bilayer structure of liposomes is appropriate for creating bio-related functions such as membrane destabilization and/or membrane fusion, promoting cellular internalization of membrane-impermeable molecules across cellular membranes into the cells.

In addition to tumor targeting, it is important to achieve controlled release so that anticancer drugs will be released preferentially in the tumor. Recently, approaches to synthesize a novel type of liposomes with low pH release feature have been reported [32,33]. Because intracellular lysosomal pH is low and also because tumor microenvironment has low pH due to hypoxic conditions [34–36], this feature provides an advantage that the drug release is more restricted to cancer cells.

In this paper, we report preparation of liposomes that efficiently encapsulate small molecule drugs and dyes and release the cargo when they encounter low pH conditions. GGTI P61A6 was efficiently encapsulated into the pH-sensitive liposomes and was delivered to human cancer cells. Inhibition of protein geranylgeranylation as well as proliferation inhibition, cell cycle effects and the accumulation of cell cycle inhibitor p21^CIP1/WAF1^ were observed by the delivery of GGTI by the pH-sensitive liposomes. We further extend the use of liposomal GGTI for cancer therapy by demonstrating that liposomal-GGTI can be combined with farnesyltransferase inhibitor (FTI) to inhibit K-Ras signaling in pancreatic cancer cells.

## Materials and Methods

### Materials

A GGTI compound P61A6 used for this study was derived from the allenoate-derived compound library as described in our previous publications [9–11]. 20 mM stock solution of GGTI P61A6 in DMSO was kept at −20°C until use. FTI compound BMS225975 was described previously [37]. This compound inhibits protein farnesyltransferase specifically with little inhibition of protein geranylgeranyltransferase I and RabGGTase. COATSOME^®^ MC-6081 (1-palmitoyl-2-oleoyl-sn-glycero-phosphocholine (POPC)), SUNBRIGHT^®^ DSPE-PG8MG (condensation product of N-(octaglycerol-glutaryl) distearoylphosphatidylethanolamine with 3-methylglutaric acid (DSPE-PG8MG)), were provided by NOF CORPORATION (Tokyo, Japan). 1, 2-dipalmitoyl-sn-glycero-3-phosphoethanolamine-N-(lissamine rhodamine B sulfonyl) (ammonium salt) (Rhodamine-PE) was purchased from Avanti Polar Lipids (Birmingham, AL, USA).

### Design and Preparation of Low pH-sensitive Liposomes

pH-sensitive empty liposomes were prepared from POPC and DSPE-PG8MG. Typical procedures were described below. POPC and DSPE-PG8MG (molar ratio; 85:15) were dissolved in a chloroform-methanol mixture, and the solvent was removed in a rotary evaporator. The lipid mixture was vacuum-dried, and hydrated with aqueous solution (10mL) containing sucrose as a cryoprotectant. After extrusion of the liposome suspension through membrane (pore size 0.22μm), the obtained suspension was freeze-dried to give the pH-sensitive empty liposomes [38].

### Rhodamine Fluorescence-labelling of Liposomes

Rhodamine-labelled pH-sensitive empty liposomes were prepared from POPC, DSPE-PG8MG, and Rhodamine-PE (molar ratio; 85:15:0.1). POPC and DSPE-PG8MG were dissolved in a chloroform-methanol mixture. Rhodamine-PE was dissolved in chloroform and added to the lipids solution, and the solvent was removed in a rotary evaporator. The lipid mixture was vacuum-dried, hydrated with phosphate buffer solution containing sucrose. After extrusion of the liposome suspension through membrane (pore size 0.22μm), the obtained suspension was freeze-dried to give the fluorescent dye-labeled pH-sensitive empty liposomes.

Rhodamine-labelled liposome powder was reconstituted in PBS buffer at 10 μg/ml, sonicated in sonicator (waterbath type) for 10 min, and added to MiaPaCa-2 cells that were cultured in 8 wells glass cell chamber for 4 hours. The cells were then washed with PBS buffer and incubated with 50 nM LysoTracker green, followed by incubation in cell culture chamber for additional 1 hour before observing with fluorescent microscope.

### Drug-loaded Liposome Preparation

A small aliquot (130 μL) of dissolved GGTI (1.2 mg) in 75% EtOH with 10% DMSO (Final concentration = 6.41 mg/ml), diluted in dH_2_O to 10%, was mixed with 1.3 mg dry powder of liposomes, and the mixture was rotated at 4°C for 4 hours followed by sonication for 2 min using a water bath-type sonicator. The liposome suspension was extruded through a polycarbonate membrane with a pore size of 100 nm. At the same time, 6 ml Sepharose 4B gel was mixed with 2 ml PBS on ice and was applied to 10 ml column, packaged at the speed of 15 cm/h. And then the mixture of liposome and GGTI was loaded on top of the gel in the column. After letting the sample to enter the resin, the column was immediately filled with cold PBS buffer, and pumping was started with a speed of 15 cm/h to remove free drugs that were not loaded into liposomes from the drug-loaded liposomes [32]. The eluents were collected and the fractions containing the liposomes were identified by their turbidity. The sample was kept at 4°C for further experiments.

### Drug and Dye Release from Liposomes

Release of drugs from liposome was measured with High-performance liquid chromatography (HPLC). 400 μl of Liposomes encapsulating GGTI (collected from Sepharose column) was mixed with 150 mM NaCl, centrifuged at 100,000 rpm for 10 min, 4C, to precipitate liposomal GGTIs. The precipitate was resuspended in 400 μl PBS. 73 μl of resuspended solution was added to 927 μl of PBS buffer with varying pH values, and then mixed at 37°C for 15 min. Triton X-100 was used as 100% control because Triton X-100 can break down the liposomes (final concentration: 0.1%). The mixtures were added to the inner tubes of ultrafilter Amicon Ultra-4 (MWCO = 10kDa, Millipore), centrifuged at 13,000 g for 10min at room temperature. The eluates were subjected to HPLC to measure the concentration of released GGTI (Waters Micromass LCT Premier Mass Spectrometer). Release of fluorescent dye Pyranine was performed as described previously [32]. A small aliquot (500 μL) of 35 mM pyranine, 50 mM DPX (p-Xylene-bis-pyridinium bromide), and 25 mM phosphate buffer solution (pH 7.4) were mixed with 7 mg of liposomes, and the mixture was sonicated for 2 min using a waterbath sonicator. Pyranine fluorescence is quenched by DPX inside of liposomes, but will emit intense fluorescence after being released from liposome. Liposomes encapsulating pyranine and DPX were added to 25 mM phosphate and 125 mM NaCl buffer with varying pH values and fluorescence intensity (512 nm) was measured with a spectrofluorometer (416 nm, Jasco FP-6500). The percent release of pyranine from liposomes was defined as Release(%) = (Ft−Fi)/(Ff−Fi)×100 where Fi and Ft mean the initial and intermediary fluorescence intensities, respectively. Ff is the fluorescent intensity after the addition of Triton X-100 (final concentration: 0.1%).

### Cell Culture

The human breast cancer cell line, MCF-7, pancreatic cancer cell line MiaPaCa2, non-small cell lung cancer cell line H596, H358, A549 and a normal human bronchial epithelium cell line BEAS-2B were obtained from the American Type Culture Collection. All cancer cell lines were maintained in DMEM or RPMI supplemented with 10% FCS (Sigma), 2% L-glutamine, 1% penicillin, and 1% streptomycin. BEAS-2B cells were cultured with BEBM media added with special growth factors (Lonza/Clonetics Co.). The medium was routinely changed every 3 days, and the cells were separated by trypsinization before reaching confluency.

### Delivery of Dyes to Cancer Cells

Doxorubicin delivery was examined as follows. After centrifuging through a Sepharose 4B column to remove free drugs, a homogeneous suspension of the doxorubicin-loaded liposomes was added to breast cancer MCF-7 cells that were cultured in an 8 well glass chamber. 6 hours after incubation, the cells were washed and examined with fluorescence microscopy to image the distribution of Doxorubicin in cancer cells. The pyranine-loaded liposomes were prepared as described above. MiaPaCa-2 cells (3 × 10^5^ cells) were cultured for overnight in an 8 wells glass cell culture chamber. Liposome suspensions were added to the cells and incubated for 4 h at 37°C. After the incubation, the cells were washed with PBS two times and observed with a fluorescent microscope (Carl Zeiss). Bafilomycin treatment was carried out with 160 nM Bafilomycin A1 for 6 hours prior to the addition of liposomes. Acridine Orange (Sigma) staining was carried out at 6 μM concentration.

### Proliferation Inhibition Assay

Proliferation inhibition assay was performed by using a cell-counting kit from Dojindo Molecular Technologies, Inc. Cancer cells were seeded in 96-well plates (5000 cells well^−1^) and incubated in fresh culture medium at 37°C in a 5% CO_2_/95% air atmosphere for 24 h. The cells were then washed with PBS and the medium was changed to a fresh medium containing GGTI-loaded liposomes or empty liposomes with same volume in buffer as control at the indicated concentrations. After 24 h, the cells were washed with PBS to remove liposomes that were not taken up by the cells, and the cells were then incubated in fresh medium for an additional 48 h. The cells were washed with PBS and incubated in DMEM with 10% WST-8 solution for another 2 h. The absorbance of each well was measured at 450 nm with a plate reader. Since the absorbance is proportional to the number of viable cells in the medium, the viable cell number was determined by using a previously prepared calibration curve (Dojindo Co.).

### Western Blot Analysis

Cells were treated with GGTI-liposomes, empty liposomes, or GGTI for 24 h, harvested, and lysed in lysis buffer (1% Triton X-100, 150 mM NaCl, 20 mM Tris-HCl at pH 7.5, 1 mM EDTA, and 1× protease inhibitor mixture). Proteins were separated by gel electrophoresis on a SDS polyacrylamide gel and then transferred to nitrocellulose membranes. The membranes were blocked with Tris-buffered saline (TBS) containing 5% (w/v) skim milk. After washing with TBS containing 0.1% Tween 20 (Sigma), the membranes were incubated overnight with the first antibody (against unprenylated form of RapI, Santa Cruz Biotechnology, CA) diluted with TBS. After washing, the membranes were incubated for 2 h with the second antibody (Santa Cruz Biotechnology, CA). Bands were detected with an ECL system (Amersham Pharmacia Biotech K.K., UK). Phosphorylation of ERK was tested using an antibody against phorphorylated form of ERK as well as against total ERK (Cell Signaling, CA). Anti-actin antibody was purchased from Sigma-Aldrich (CA).

### Statistical analysis

All results are expressed as means ± SD. Statistical comparisons were made using Student’s t-test after analysis of variance. The results were considered to be significantly different at P value < 0.05, marked with *.

## Results

### Design and Preparation of Low pH-sensitive Liposomes (pH-liposomes)

**Fig 1:**
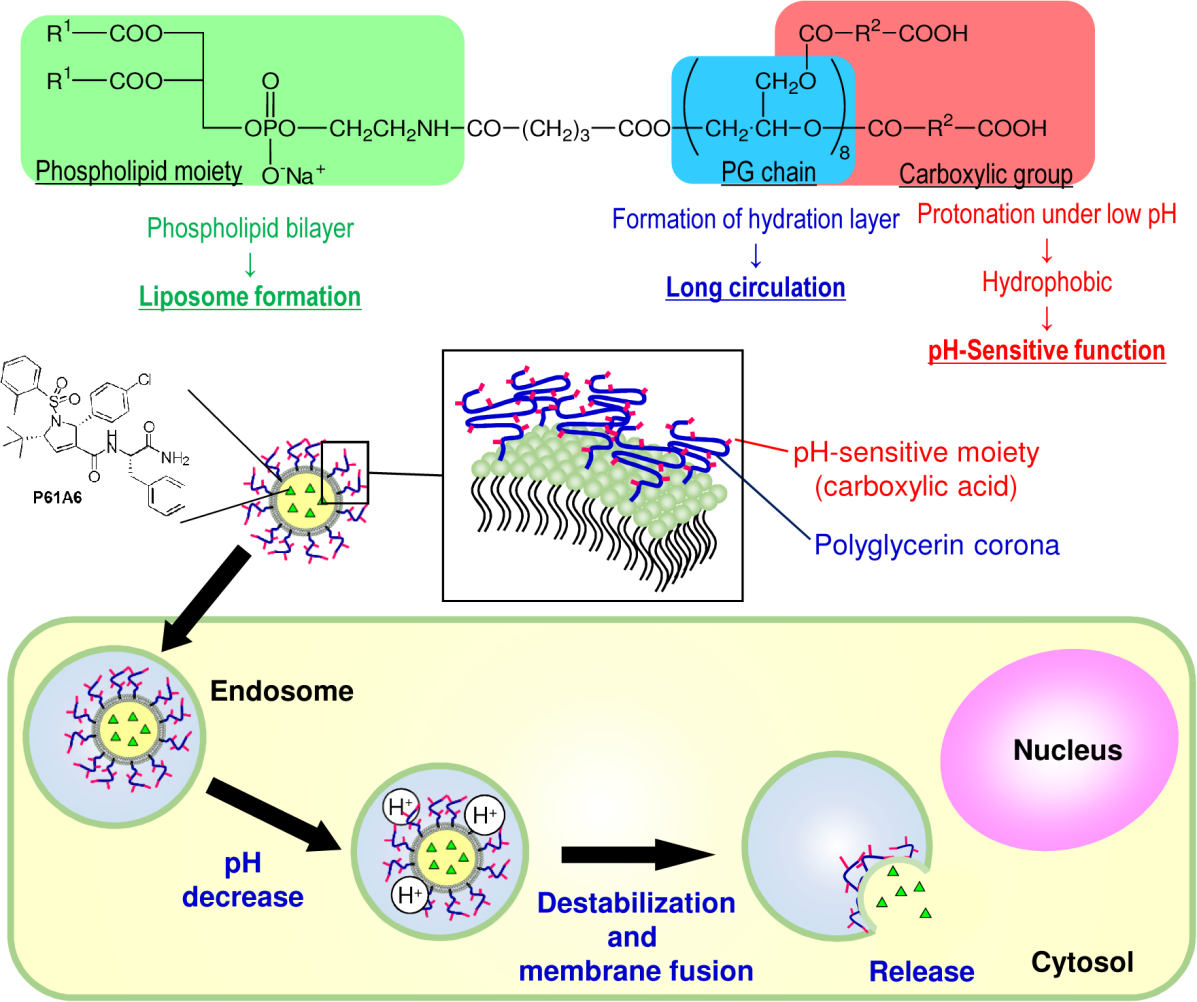
The scheme of synthesis of pH-responsive liposome and the proposed intracellular drug release pathway.

Our pH-liposomes are stable at physiological pH, but become destabilized under acidic conditions due to protonation, therefore responding to low pH environments of endosome-lysosomes inside the cells [32]. The liposomes were prepared by using two different lipids, DSPE-PG8MG and POPC. A general scheme of DSPE-PG8MG is shown in Fig 1. This molecule contains a phospholipid that is linked to carboxylated polyglycerin moiety (PG chain). Under acidic conditions, the PG chain is protonated resulting in destabilization of liposomal membranes causing fusion with endosomal membranes. The content of liposomes will then be released into the cytosol. By changing the ratio of the two lipids, it is possible to design liposomes that respond to different pH values. Here, the polyglycerin modified with 3-methyl glutaric acid is known to have a pKa value of 6.3 [39,40]. Therefore, DSPE-PG8MG has a similar pKa value of the polyglycerin derivative, and will have a pH-responsiveness near pH 6. In our case, we wanted to achieve content release at pH below 6 but little release above pH 6. Thus, it was decided to find an appropriate lipid composition in the similar procedure. Briefly, dispersions of pH-liposomes which encapsulated pyranine dye were added to solutions at various pH values. And the pH-responsiveness of the liposomes was evaluated by measuring the amount of pyranine released from them. After testing different ratios, we decided to use the POPC and DSPE-PG8MG ratio of 85 and15 (mol%).

### Intracellular Localization of pH-liposomes

**Fig 2:**
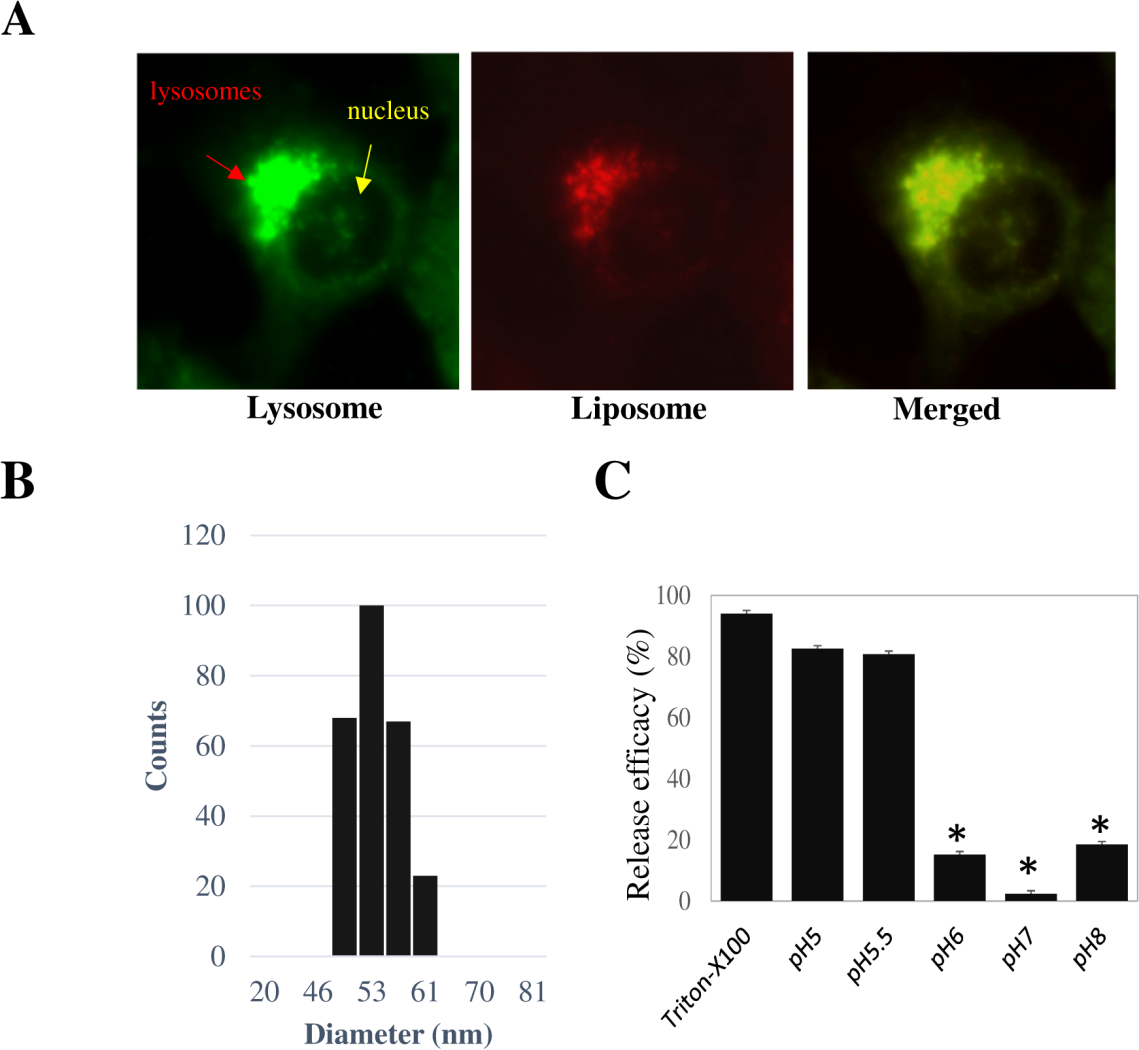
A: Fluorescent microscopy images of MiaPaCa-2 cells treated with Rhodamin-labeled liposomes for 4 hours. Green fluorescence shows lysosomes, stained with LysoTracker (Life Technologies). Red fluorescence shows Rhodamin-labeled liposomes. B: Dynamic light scattering of GGTI-loaded liposome is shown. GGTI-loaded liposomes were prepared as described in Materials and Methods. C: Low pH release of GGTI from GGTI-loaded liposomes. GGTI-loaded liposomes were prepared and then exposed to solution adjusted to different pH values for 15 min and the release of GGTI was examined by HPLC. TritonX-100 was used as a full release control. The symbol * indicates statistically significant difference at P value < 0.05.

To investigate cellular uptake and intracellular localization of pH-liposomes, we synthesized Rhodamine-labelled pH-sensitive empty liposomes (Rhodamine dye was covalently conjugated to the lipids during synthesis) as described in the Materials and Methods section. Pancreatic cancer cells MiaPaCa-2 were incubated with Rhodamine-labelled liposomes for 4 hours and liposomal fluorescence was examined with a fluorescent microscope. As shown in Fig 2A, punctate fluorescence was observed at a perinuclear region indicative of lysosomal localization. This point was confirmed by the use of Lysosensor Green DND-189 (Life Technologies). Lysosensor Green DND-189 exhibits green fluorescence only when inside intracellular acidic compartments such as lysosomes. Co-localization of red Rhodamin fluorescence and green Lysosensor fluorescence was observed, confirming lysosomal localization of the liposomes. Thus, liposomes appear to accumulate in lysosomes when taken up by cells.

### Drug Loading and Release

Liposome’s loading efficiency of drugs and dyes was tested by mixing with liposomes, using Sepharose 4B column to remove free drugs and releasing the content from liposomes by Triton X-100. GGTI concentration was measured by comparing with a standard GGTI sample using LC-MS. We estimated that the loading capacity of GGTI into liposomes is approximately 30% of total GGTI. To examine the size of reconstituted liposomes, we subjected GGTI-loaded liposomes to dynamic light scattering (DLS) measurement. As can be seen in Fig 2B, we found that the size of the GGTI-loaded liposomes ranged from 46 to 65 nm in diameter with average size of 54.9 nm. Thus, our preparation has a relatively narrow size distribution.

Release of GGTI from liposomes was examined by loading GGTI, collecting GGTI-loaded liposomes and releasing the content by exposing to PBS buffer with various pH values ranging from 4.5 to 8. As shown in Fig 2C, liposomes retained GGTI inside tightly at neutral or basic buffers. Little release of GGTI was observed at a pH range 6–8. However, when pH was below 6, a significant release of GGTI from liposomes was observed. At pH 5.5, more than 80% of loaded GGTI was released. Similarly, almost complete release was observed at pH 5 and at pH 4.5 (not shown). These results suggest that the pH-sensitive liposomes were efficiently destabilized at acidic conditions. A sharp transition between pH 5.5 and pH 6 suggests that this type of liposome provides a suitable vehicle for intracellular controlled release.

### Low pH-dependent Delivery of Loaded Dye Inside the Cell

**Fig 3:**
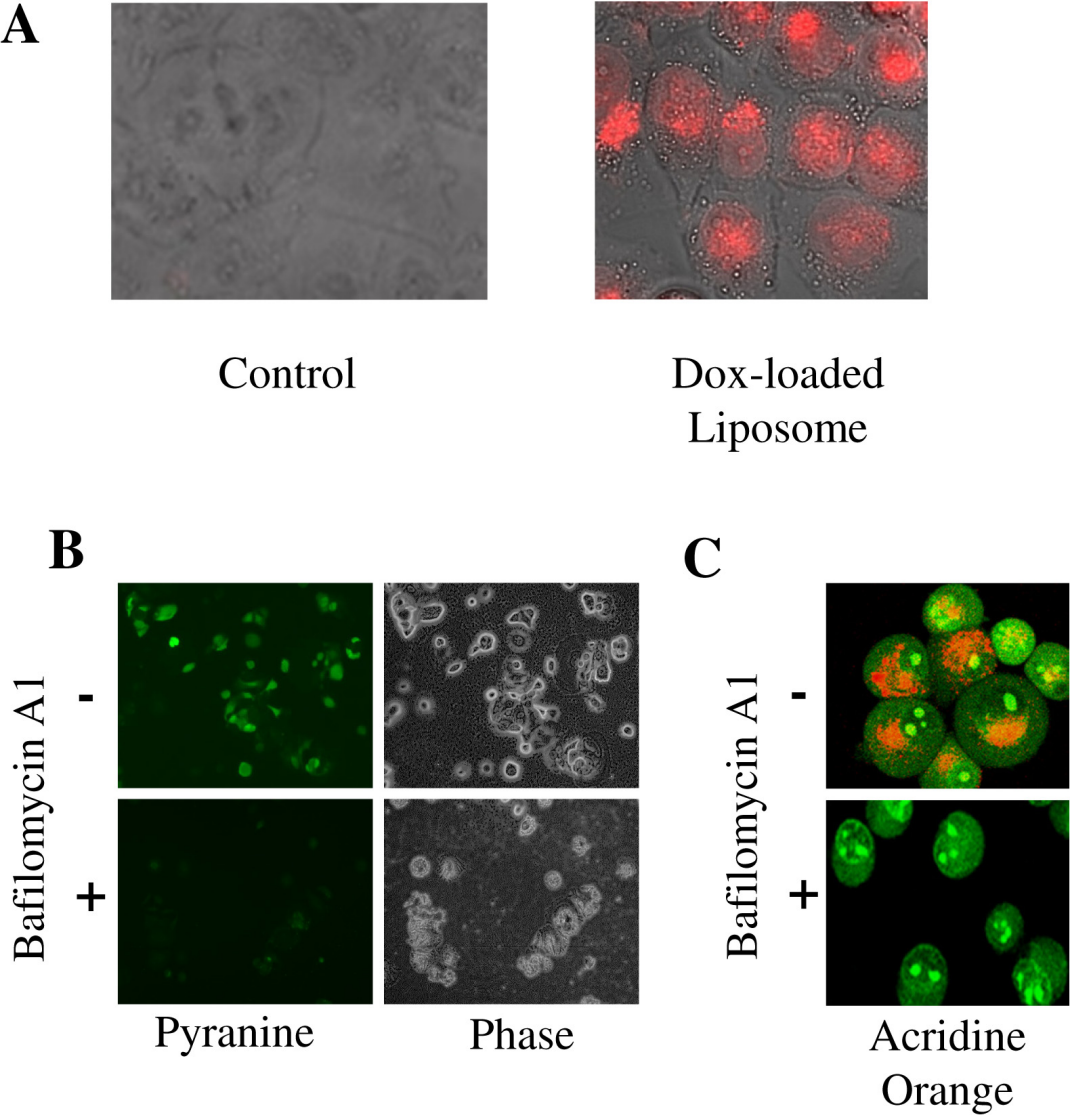
Release of dye and drug from liposome is dependent on low pH of acidic organelles. **A**: Fluorescent microscopy images of MCF7 cells incubated with Doxorubicin-loaded liposomes for 6 hours (red fluorescence). Control cells did not receive liposomes. **B**: Release of Pyranine dye in MiaPaCa-2 cells is examined by its fluorescence with or without the treatment with Bafilomycin A1. Phase contrast picture is shown. **C**: Effects of Bafilomycin A1 on pH of intracellular organelles are shown using Acridine Orange.

The doxorubicin loaded liposomes were used to examine intracellular drug release capability, since doxorubicin emits strong red fluorescence under UV excitation. After eluting from a Sepharose 4B column to remove free drugs, a homogeneous suspension of the doxorubicin-loaded liposomes was added to breast cancer MCF-7 cells that were cultured in an eight-well glass chamber. 6 hours after incubation, the cells were washed and examined with fluorescence microscopy to image the distribution of Doxorubicin in cancer cells. The cells treated with doxorubicin showed strong red fluorescence (Fig 3A) in the cytosol and in nuclei. This was similar to that observed with cells treated with free doxorubicin (data not shown). On the other hand, control cells treated with liposomes without doxorubicin remained non-fluorescent.

The above experiments show that liposomes can be loaded with dyes and drugs and successfully and effectively deliver the content to cancer cells. We wanted to further characterize the low pH-dependent release feature of the liposomes inside the cell. To investigate this point, we used Pyranine dye loaded liposomes and treated cells with Bafilomycin, a drug that alters lysosomal pH. Bafilomycin A1 (Baf) is a specific inhibitor of the intracellular vacuolar proton pump, inhibiting acidification of the vacuolar system, such as endo/lysosomes, thus increase lysosomal pH [41,42]. Pancreatic cancer cells MiaPaCa-2 were treated with 160 nM Baf for 6 hours and then Pyranine dye-loaded liposomes were added to the cells. The cells were incubated for an additional 12 hours before examination by fluorescence microscopy. As shown in Fig 3B, the treatment with Baf completely prevented intracellular release of Pyranine, demonstrated by the lack of staining, as compared to the bright green fluorescence of the cells unexposed to Baf. To confirm the effect of Baf on the de-acidification of endosomes, acridine orange (AO) staining was performed. AO is a fluorescent weak base that is frequently used as a probe for monitoring the acidification of organelles. In neutral or alkali environments this dye emits green fluorescence, but when exposed to acidic compartments, it is ionized and its emission undergoes a red shift. In the untreated cells, red fluorescence was observed inside discrete cytoplasm organelles, indicating that the AO had accumulated in acidic organelles (Fig 3C). However, the red fluorescence dramatically decreased after 6 hours of Baf treatment, indicating that the Baf had increased the pH of the endosomes in the MiaPaca-2 cells. These results suggest that the release of dye from the liposomes is dependent on low pH conditions in intracellular organelles.

### Liposomal GGTI Inhibits Protein Geranylgeranylation Inside the Cell and This Effect is Dependent on Low pH of Lysosomes

**Fig 4:**
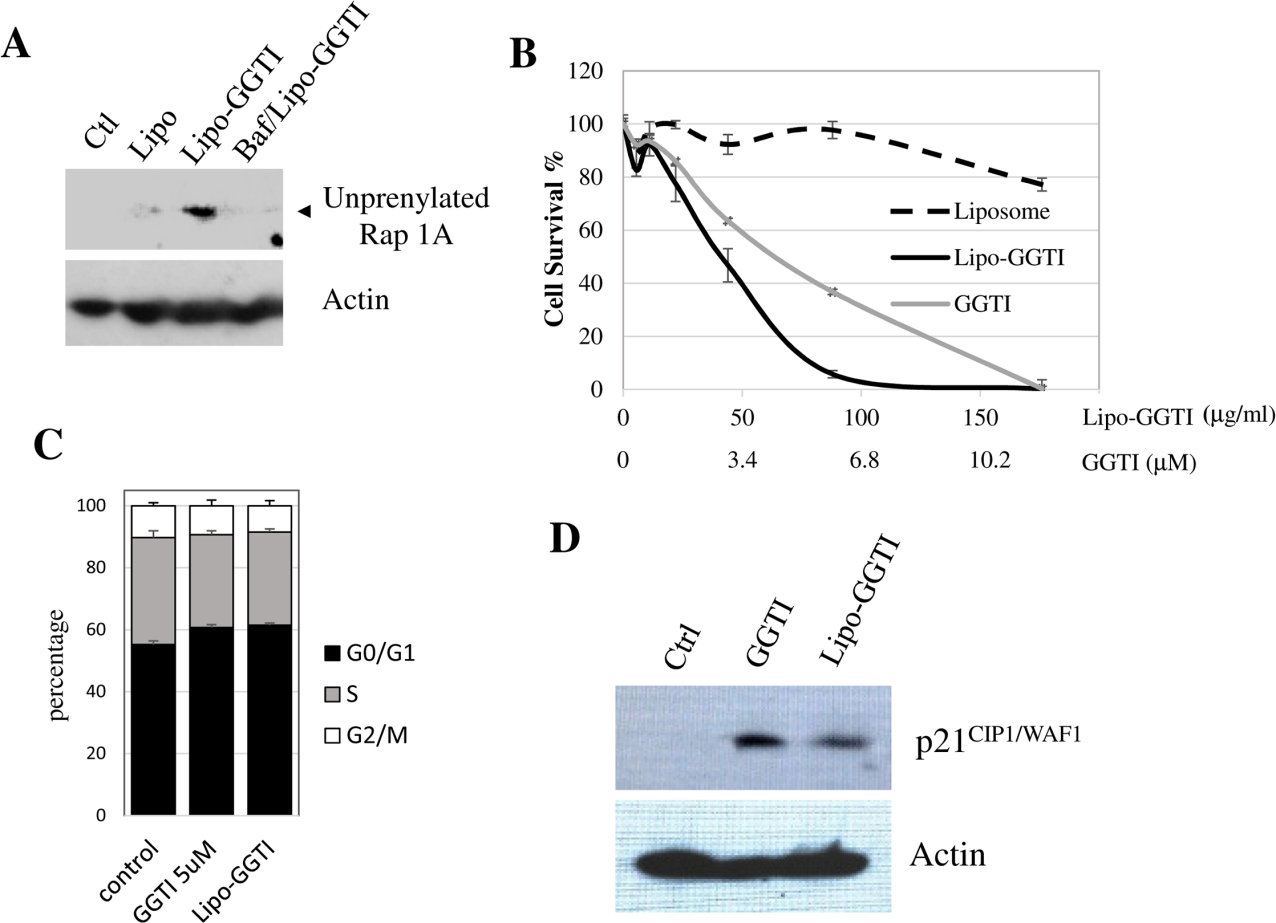
A: Inhibition of protein geranylgeranylation by liposomal GGTI was examined by the use of an antibody that specifically detects unprenylated form of Rap1A. MiaPaCa-2 cells were treated for 3 hours with GGTI-loaded liposomes with or without Bafilomycin pretreatment and the amount of unprenylated Rap1A was examined by Western. Lipo: empty liposome. Lipo-GGTI: GGTI loaded liposome. Baf/Lipo-GGTI: GGTI loaded liposome was added to cells pretreated with Bafilomycin A. B: Cell proliferation inhibition by GGTI-loaded liposomes after 72 hour treatment. Empty liposomes and free GGTI are used as comparison. C: Cell cycle effect of liposomal-GGTI (Lipo-GGTI) was examined by FACS analysis after treating MiaPaCa-2 cells with liposomal-GGTI for 24 hours. Similar level of G1 accumulation was observed with that seen with free GGTI. D: Effect of liposomal-GGTI on p21^CIP1/WAF1^ expression was examined by Western analysis using MiaPaCa-2 cells treated with liposomal-GGTI for 24 hours.

GGTI release and efficacy to inhibit protein geranylgeranylation inside cancer cells were investigated. Fig 4A shows that the delivery of GGTI by the liposomes results in the inhibition of protein geranylgeranylation inside the cell. In this experiment, MiaPaCa-2 cells were treated with liposomal-GGTI for 3 hours, and proteins were collected from cell lysate for Western analysis. As shown, treatment of cells with either GGTI P61-A6 alone or GGTI-loaded liposome led to the appearance of unprenylated Rap1 band, indicating that liposomes deliver and release GGTI compound inside of cells to function and inhibited protein geranylgeranylation. Since the pH-liposomes exhibit significant destabilization below pH 6, which corresponds to pH of endolysosome interior, the pH-liposomes were likely to be destabilized or fused with endosomal membranes resulting in the release of the drug cargo into cytosol, inhibiting GGTase-I and blocking Rap1 prenylation. To examine the pH-sensitivity of GGTI release, the same experiment was repeated after treating the cells with pH-altering compound Bafilomycin A1. As shown in Fig 4A, treatment with Bafilomycin A1 abolished the effect of liposomal-GGTI on Rap1 prenylation. This is in line with the idea that increased pH of lysosomes altered by Bafilomycin A1 could not destabilize pH-liposomes anymore, therefore, GGTIs were kept inside of liposomes. Therefore, when taken up by the cells, the acidity of endosomes is critical for destabilization and/or fusion of pH-liposomes with endolysosome for efficient release and transfer of the contents into cytosol.

### Liposomal GGTI Causes Inhibition of Proliferation of Pancreatic and Lung Cancer Cells

The effect of drug delivery from pH-liposomes was examined with cell proliferation assay. Pancreatic cancer cell line MiaPaCa-2 was treated with unloaded liposomes, GGTI-loaded liposomes or free GGTI (same volume in buffer) with various concentrations for 72 hours. As shown in Fig 4B, significant inhibition of proliferation was observed with GGTI-loaded liposome. This inhibition was similar or better than that seen with free GGTI. In contrast, empty liposomes did not affect cell proliferation even at 180 μg/mL. The liposomes themselves appear to be non-toxic at these concentrations.

One of the main features of GGTI is that they induce cell cycle arrest at G1 phase. To examine whether this applies to cell effects using liposomal GGTI, cells treated with liposomal GGTI were analyzed by Flow cytometry. As shown in Fig 4C, treatment of MiaPaca-2 cells with either GGTI solution or Lipo-GGTI for 24 h caused enrichment of G1 phase cells, while the percentage of S-phase cells was reduced by these treatments. Percentage of G2 cells was changed only slightly.

We have previously shown that GGTI induces expression of a cell cycle regulator p21^CIP1/WAF1^ [7]. Induction of this cyclin-dependent kinase inhibitor results in the accumulation of G1 phase cells. Our previous studies showed that RhoA is one of the main targets of GGTI and that RhoA suppresses p21^CIP1/WAF1^ expression. To examine whether liposomal-GGTI induces p21^CIP1/WAF1^ expression, we treated MiaPaCa-2 cells with liposomal GGTI and carried out Western analysis. As shown in Fig 4D, significant induction of p21^CIP1/WAF1^ was observed by the treatment with liposomal GGTI.

**Fig 5:**
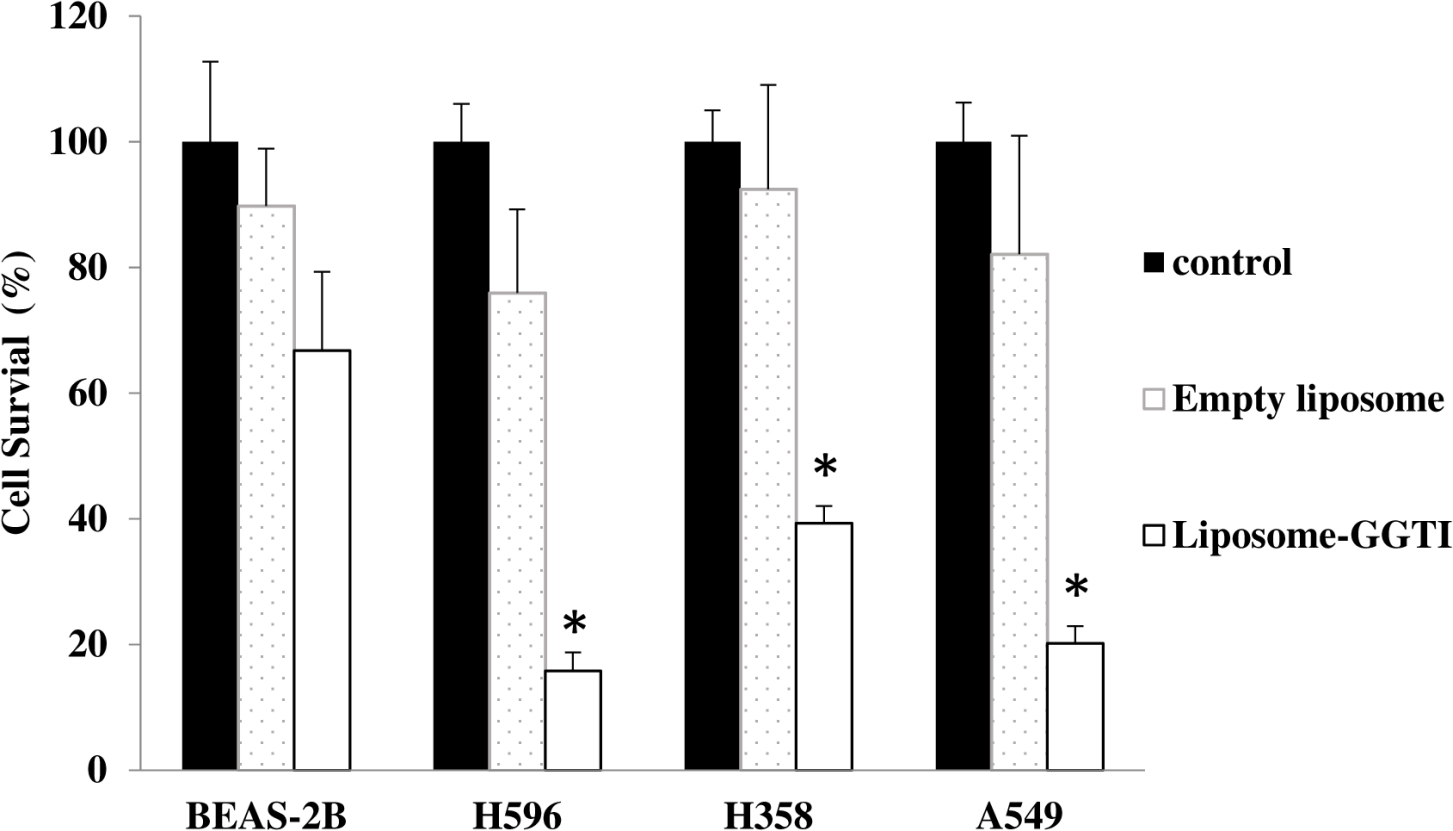
Cell proliferation inhibition of GGTI-loaded liposomes was examined with human non-small cell lung cancer cells H596, H358 and A549 as well as a normal lung cell. Cell numbers after 72 hours’ treatments are shown as percentage of untreated cells. Empty liposomes do not affect proliferation. The symbol * indicates statistically significant difference at P value < 0.05.

In our previous studies, we showed that GGTI P61A6 inhibited both pancreatic and lung cancer cells in animal models, therefore, we also tested liposomal GGTI with different lung cancer cell lines, H596, H358 and A549 as well as with normal bronchial epithelial cell line BEAS-2B. As shown in Fig 5, compared to unloaded liposomes, liposome-GGTI showed significant cell proliferation inhibition with a variety of non-small lung cancer cell lines tested. Liposomal GGTI suppressed approximately 60–80% of cell proliferation, while the same concentration of empty liposomes did not. Interestingly, the normal human bronchial epithelium cell line BEAS-2B showed unexpectedly strong resistance to liposomal GGTI compared with lung cancer cells. The mechanism for this resistance remains unknown and warrants further investigation.

### Liposomal GGTI Synergizes with FTI (Farnesyltransferase inhibitor) to Inhibit Proliferation of K-Ras Activated Cancer Cells

The above results suggest that liposomal-GGTI is a novel type of anticancer drugs that has the potential to be delivered to tumor. This opens up the possibility that it can be used to inhibit K-Ras signaling that is activated in a number of human cancer cases including pancreatic, lung and colon cancers. The Ras family proteins, especially K-Ras, can be alternatively prenylated by either FTase or GGTase-I [43–45]. Thus, inhibition of either enzyme cannot achieve complete inhibition of protein lipid modification. But simultaneous inhibition of both enzymes can lead to complete inhibition of K-Ras prenylation and signaling [46]. This, however, is not easy to achieve with free drugs, as FTI/GGTI combination can cause toxicity to other normal tissues, because Ras family is critical for a number of cellular functions [47]. Therefore, preferential and exclusive delivery of GGTI to tumor by using liposomes will allow us to combine these two types of compounds at the same time for cancer treatment without severe side effects to noncancerous tissues.

**Fig 6:**
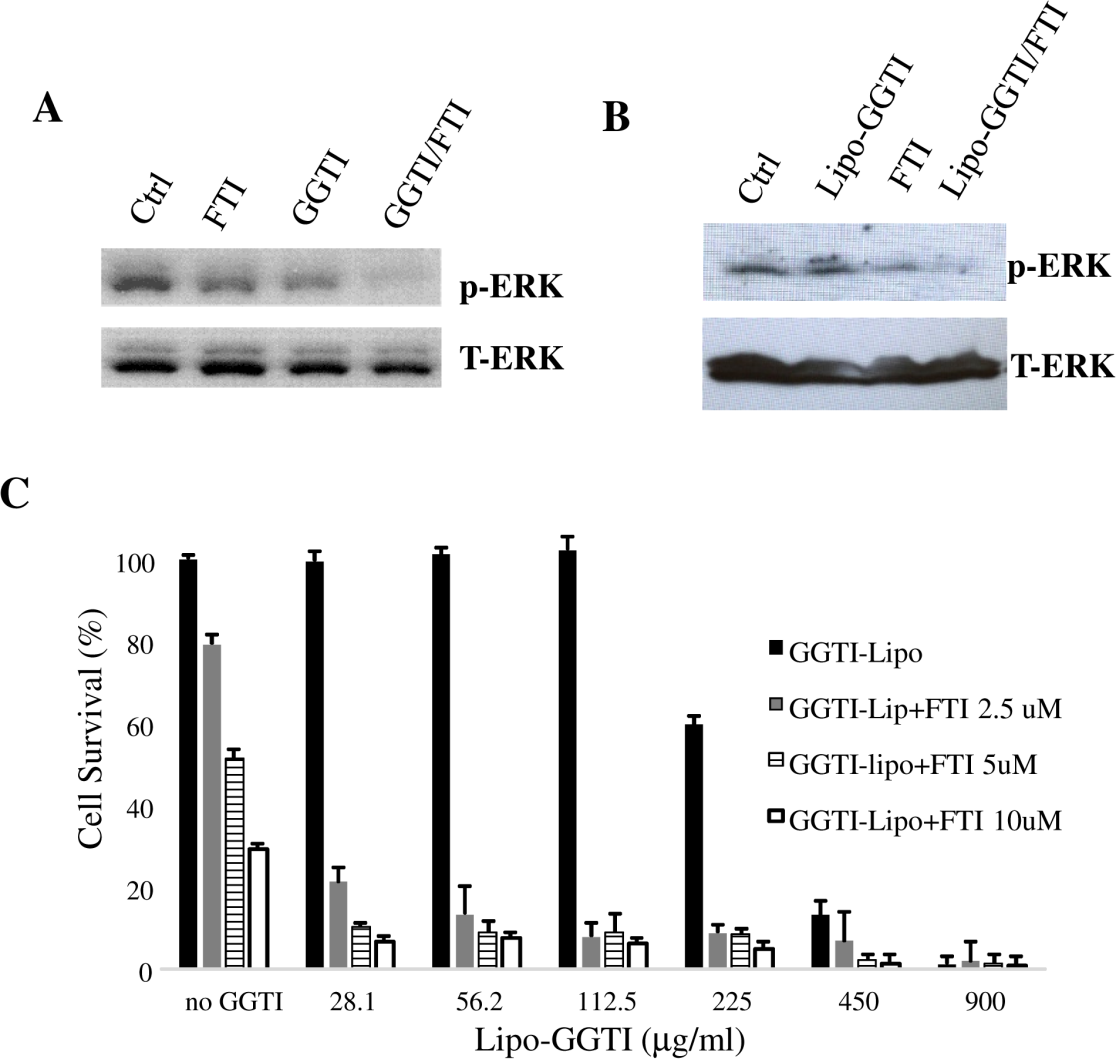
Effect of combination of liposomal GGTI and FTI on MiaPaCa-2 cells. **A**: Effects of GGTI or FTI alone as well as the combination of the two on ERK phosphorylation were examined by Western blot after 12 hours of treatment. Total ERK is used as loading control. **B**: the same experiment was repeated with liposomal GGTI (Lipo-GGTI) and FTI. **C:** Effects of the combination of liposomal-GGTI and FTI on proliferation of MiaPaCa-2 cells after the treatment for 48 hours. FTI and liposomal GGTI concentrations are varied.

To explore the possibility of combining liposomal-GGTI with FTI, we treated pancreatic cancer cells MiaPaCa-2 with liposomal-GGTI and FTI for 12 hours and examined its effect on ERK phosphorylation. As shown in Fig 6B, combination of FTI and Liposomal-GGTI led to complete inhibition of ERK phosphorylation in cancer cells, while treatment with either Liposomal-GGTI or FTI alone only partially inhibited ERK phosphorylation. The total amount of ERK was unaffected by these treatments. These results are similar to those obtained using free drug combinations, GGTI and FTI (Fig 6A).

Effects of the liposomal-GGTI/FTI combination on cell proliferation after 48 hours of treatment were shown in Fig 6C. We observed that FTI addition to liposomal-GGTI resulted in significant suppression of cell proliferation. 28 μg/ml Liposome-GGTI combined with 2.5 μM of FTI suppressed cell proliferation up to approximately 80%, although either single compound of this concentration did not show significant effect. Combination Index was calculated to be 0.5102. Thus, synergistic effects can be observed.

## Discussion

Geranylgeranyltransferase inhibitors (GGTI) have defined a type of anticancer drugs that act to inhibit membrane association of signaling proteins such as Rho proteins. These compounds have shown promising anticancer activity [10,11,48–50]. In this paper, we report preparation of a new generation of GGTI that is encapsulated into liposomes. We were successful in achieving sufficient loading of the drug to liposomes. The encapsulated GGTI was delivered to human cancer cells resulting in inhibition of protein geranylgeranylation and exhibiting cellular effects expected from this type of drug. Thus, liposomal GGTI provides a new reagent that could be developed for cancer therapy.

One of the attractive features of the liposomal-GGTI reported here is that GGTI is released by exposure to low pH. To design the liposomes, we adjusted the ratio of two lipids that constitute our liposomes. One of the lipids, polyglycerin-phospholipids, contains carboxylated polyglycerin and protonation of the carboxylated polyglyerin portion results in destabilizing the liposome, possibly facilitating membrane fusion leading to the release of the content or releasing the drugs in lysosomes and subsequently delivering into cytosol. This lipid is mixed with another lipid, palmitoyl oleoyl PC, and the ratio of the two lipids determines that release pH. We have adjusted the ratio so that the release occurs at pH values below 6. We then established that the liposomes accumulate in endosome/lysosomes by following the cellular localization of fluorescent liposomes.

We provided experimental evidence that the delivery of GGTI into cancer cells is via a low-pH dependent mechanism. This was accomplished by using Bafilomycin A1, an inhibitor of proton pump on endosomes/lysosomes. We have observed inhibition of protein geranylgeranylation inside the cell by liposomal GGTI and this effect was blocked by raising the endosomal/lysosomal pH by the treatment with Bafilomycin A1. Similarly, delivery of a dye Pyranine by using this liposome was blocked by treating cells with Bafilomycin. By altering the ratio of two lipids, it will be possible to further adjust the releasing pH. For example, we may be able to tailor our liposomes to release drugs under various pH conditions that exist among different cancer cell types.

The liposomal GGTI exerts cellular effects that were expected of GGTI effects [7]. Liposomal GGTI inhibited proliferation of a pancreatic cancer cell line MiaPaCa-2 and this proliferation inhibition was associated with the accumulation of G1 phase cells with reduction of S phase cells. In addition, induction of a cell cycle regulator, p21^CIP1/WAF1^ was observed. These cellular effects are hallmarks of GGTI cellular effects and the results further confirm that GGTI was successfully delivered to cancer cells. It is important to point out that the liposomes themselves without GGTI appear to have little effects on cell proliferation. Thus, the liposomes we used appear to be biocompatible.

The availability of liposomal-GGTI opens up the possibility to combine GGTI with other anticancer drugs. Of particular interest is farnesyltransferase inhibitor (FTI) that has been extensively studied in the past [51–53]. FTI has been developed as anticancer drugs to inhibit Ras proteins that are mutated in a wide range of human cancers [54], pancreatic cancer and lung cancer in particular. However, it was later found that, while K-Ras and N-Ras are normally farnesylated, they can be alternatively modified by geranylgeranylation [43–45]. Attempts to combine FTI and GGTI as well as to use dual inhibitors of FTase and GGTase-I have been made in the past [1,47,55]. However, these experiments failed because sufficient concentration of the drugs to inhibit K-Ras was never achieved mainly due to cytotoxic effects of inhibiting both farnesylation and geranylgeranylation. Our development of liposomal GGTI raises the possibility that the combined effects of free FTI and liposomal GGTI can be accomplished only in the tumor. As a first step to investigate this possibility, we have combined our liposomal GGTI and free FTI together. We demonstrated that the activation of ERK can be accomplished by the combination of the two drugs but not each. In addition, we found that the liposomal GGTI/free FTI combination inhibits proliferation of pancreatic cancer cells and that synergistic effects were observed. Further experiments are needed to investigate the efficacy of the liposomal GGTI/free FTI combination.

In summary, we have designed and developed a new generation of GGTI that is encapsulated in nanoparticles. They can deliver GGTI into human cancer cells and cause cellular effects. In addition, they can be combined with free FTI to inhibit the K-Ras signaling in human cancer cells. Currently, we are testing biodistribution of our pH-sensitive liposomes using mice with pancreatic cancer xenograft. Preliminary experiments to Intravenous inject the pH-sensitive liposomes (fluorescent) and examining fluorescence in the tumor, liver, kidney and lung revealed significant accumulation of the liposome in the tumor (J.L. and F.T., unpublished results). In addition to the liposomal-GGTI described here, we have also succeeded in encapsulating GGTI into liposomes that have transferrin attached (J.L. and F.T., unpublished results). Since transferrin receptor is overexpressed in cancer cells, these particles can be targeted to cancer cells by using this active targeting in addition to the passive EPR-mediated targeting. A variety of nanoparticle encapsulated GGTI can be developed in the future.
